# Cost-effectiveness analysis of a multiple health behaviour change intervention in people aged between 45 and 75 years: a cluster randomized controlled trial in primary care (EIRA study)

**DOI:** 10.1186/s12966-021-01144-5

**Published:** 2021-07-02

**Authors:** Ignacio Aznar-Lou, Edurne Zabaleta-Del-Olmo, Marc Casajuana-Closas, Alba Sánchez-Viñas, Elizabeth Parody-Rúa, Bonaventura Bolíbar, Montserrat Iracheta-Todó, Oana Bulilete, Tomàs López-Jiménez, Haizea Pombo-Ramos, María Victoria Martín Miguel, Rosa Magallón-Botaya, Jose Ángel Maderuelo-Fernández, Emma Motrico, Juan Bellón, Ruth Martí-Lluch, Maria Rubio-Valera, Antoni Serrano-Blanco

**Affiliations:** 1 Research and development Unit, Parc Sanitari Sant Joan de Déu, Institut de Recerca Sant Joan de Déu, Dr. Antoni Pujades 42, 08830, Sant Boi de Llobregat, Barcelona, Catalonia Spain; 2 Consortium for Biomedical Research in Epidemiology & Public Health (CIBER en Epidemiología y Salud Pública - CIBERESP), Madrid, Spain; 3 Fundació Institut Universitari per a la recerca a l’Atenció Primària de Salut Jordi Gol i Gurina (IDIAPJGol), Barcelona, Spain; 4 Gerència Territorial de Barcelona, Institut Català de la Salut, Barcelona, Spain; 5 Departament d’Infermeria, Facultat d’Infermeria, Universitat de Girona, Girona, Spain; 6 Universitat Autònoma de Barcelona, Bellaterra, Cerdanyola del Vallès Spain; 7 Primary Care Prevention and Health Promotion Network (redIAPP), Palma de Mallorca, Spain; 8 Primary Care Research Unit, Mallorca, Balearic Public Health Service, Palma de Mallorca, Spain; 9 Health Research Institute of the Balearic Islands (IdISBa), Palma de Mallorca, Spain; 10 Primary Care Research Unit of Bizkaia, Basque Health Service-Osakidetza, Bilbao, Spain; 11 Biocruces Bizkaia Health Research Institute, Barakaldo, Bizkaia Spain; 12 Vigo Primary Health Care, Vigo, Spain; 13 I-Saúde Research Group (IISGS), Vigo, Spain; 14 IIS-Aragón Grupo b21-17R, Universidad de Zaragoza, Zaragoza, Spain; 15 CS Arrabal.Servicio Aragonés de Salud, Zaragoza, Spain; 16 Primary Health Care Research Unit of Salamanca (APISAL), Health Service of Castilla y León (SACyL), Institute of Biomedical Research of Salamanca (IBSAL), Salamanca, Spain; 17 Universidad Loyola Andalucía, Sevilla, Spain; 18 Centro de Salud El Palo, Málaga, Spain; 19 Department of Preventive Medicine, University of Málaga, Málaga, Spain; 20 Biomedical Research Institute of Malaga (IBIMA), Málaga, Spain; 21 ISV Research Group, Research Unit in Primary Care, Primary Care Services, Girona, Catalan Institute of Health (ICS), Girona, Catalonia Spain; 22 Biomedical Research Institute, Girona (IdIBGi), ICS, Girona, Catalonia Spain

**Keywords:** Economic evaluation, Health promotion, Primary care, Hybrid trial

## Abstract

**Background:**

Multiple health behaviour change (MHBC) interventions that promote healthy lifestyles may be an efficient approach in the prevention or treatment of chronic diseases in primary care. This study aims to evaluate the cost-utility and cost-effectiveness of the health promotion EIRA intervention in terms of MHBC and cardiovascular reduction.

**Methods:**

An economic evaluation alongside a 12-month cluster-randomised (1:1) controlled trial conducted between 2017 and 2018 in 25 primary healthcare centres from seven Spanish regions. The study took societal and healthcare provider perspectives. Patients included were between 45 and 75 years old and had any two of these three behaviours: smoking, insufficient physical activity or low adherence to Mediterranean dietary pattern. Intervention duration was 12 months and combined three action levels (individual, group and community). MHBC, defined as a change in at least two health risk behaviours, and cardiovascular risk (expressed in % points) were the outcomes used to calculate incremental cost-effectiveness ratios (ICER). Quality-adjusted life-years (QALYs) were estimated and used to calculate incremental cost-utility ratios (ICUR). Missing data was imputed and bootstrapping with 1000 replications was used to handle uncertainty in the modelling results.

**Results:**

The study included 3062 participants. Intervention costs were €295 higher than usual care costs. Five per-cent additional patients in the intervention group did a MHBC compared to usual care patients. Differences in QALYS or cardiovascular risk between-group were close to 0 (-0.01 and 0.17 respectively). The ICER was €5,598 per extra health behaviour change in one patient and €1,727 per one-point reduction in cardiovascular risk from a societal perspective. The cost-utility analysis showed that the intervention increased costs and has no effect, in terms of QALYs, compared to usual care from a societal perspective. Cost-utility planes showed high uncertainty surrounding the ICUR. Sensitivity analysis showed results in line with the main analysis.

**Conclusion:**

The efficiency of EIRA intervention cannot be fully established and its recommendation should be conditioned by results on medium-long term effects.

**Trial registration:**

Clinicaltrials.gov NCT03136211. Registered 02 May 2017 – Retrospectively registered

**Supplementary Information:**

The online version contains supplementary material available at 10.1186/s12966-021-01144-5.

## Introduction

Chronic diseases are one of the main challenges for health systems. These diseases account for a high proportion of morbidity and mortality worldwide [1]. The cost of these diseases, when they are considered alone, has a relevant impact but it is even higher when comorbidities are present [2]. It is estimated that 10 million deaths attributed to chronic diseases can be prevented by adopting healthy behaviours while not adopting them has a significant economic impact [3, 4]. Against this background, the primary health care (PHC) system offers comprehensive, continuous care as the optimal context for the promotion of healthy lifestyles [5]. Health promotion does not have to be focused on specific health risk, but it can address several of them together. In this context, multiple health behaviour changes (MHBC) interventions approach several risk factors within the same intervention [6], which might increase their efficiency and effectiveness.

However, as far as we know, few economic evaluations have assessed the impact of MHBC interventions [7]. *McRobbie* et al showed that a diet and physical activity intervention addressing weight management in obese patients had an incremental cost-utility ratio (ICUR) of £7400 per quality-adjusted life-year (QALY) gained using a health system perspective [7], while van *Keulen* et al showed that a tailored print intervention aiming to improve physical activity and/or diet in adults between 45 and 70 years cost €2867 per QALY gained and €160 per improved behaviour [8]. Similar evidence is available on the assessment of single behaviour interventions. Systematic reviews focused on cardiovascular risk (CVR) or physical activity did not find a great number of economic evaluations based on randomized clinical trials [9, 10]. Indeed, some authors have pointed out that more robust, real-world evidence focused on MHBC intervention is needed, especially in light of the resource requirements to implement them [9].

The EIRA study contained an MHBC intervention focused on smoking cessation, physical activity and adherence to the Mediterranean dietary pattern in PHC patients [11]. This study aimed to evaluate the cost-utility and cost-effectiveness, in terms of MHBC and CVR reduction, of the EIRA intervention through a cluster-randomized controlled trial.

## Methods

This was an economic evaluation alongside a 12-month cluster-randomized control trial conducted between 2017 and 2018 in 25 PHC centres from seven Spanish regions. The cost perspectives taken were societal and healthcare providers. The study protocol (NCT03136211.R), with details on the study design and intervention, was published elsewhere [11].

### Setting

The Spanish public healthcare system provides universal coverage for citizens and foreign nationals. It is funded through taxes and free of charge at the point of use, with some exceptions such as medication. Although it is a decentralised system, where each of the 17 Spanish regions controls health planning, public health and the management of health services (including tariffs publication), PHC is the most accessible point of contact within the public system [12].

### Participants

#### PHC centres

The following inclusion criteria were used for selection: 1) internet access; 2) possibility of developing community activities; 3) not being located in areas with high cultural or linguistic diversity or tourist areas and; 4) having a signed commitment document from the management team. Healthcare professionals and administrative staff were involved in the study and signed a collaboration commitment form.

Participants were people aged between 45 and 75 years old who engaged in at least two of these behaviours: smoking, insufficient physical activity or low adherence to Mediterranean dietary pattern. Participants had to be registered in the health system and have an assigned healthcare professional. They needed to provide signed informed consent. The exclusion criteria set were: advanced serious illnesses, dependence in daily life activities, being included in long-term home health programme and planning to move to another area in the current year.

### Randomisation

Twenty-six PHC centres were computer randomized 1:1 based on each region at a central location (IDIAP Jordi Gol, Barcelona, Spain). After randomisation, one intervention centre dropped out due to administrative reasons, leaving 12 centres in the intervention group. Participants were assigned to the intervention or control group based on their PHC centre.

### Interventions

EIRA was a health promotion intervention designed following Medical Research Council methodology [13] (including systematic reviews [14–22], citizen and professional participation through qualitative research [23–25] and a previous pilot study, among other techniques) and was based on the Transtheoretical Model [26]. Physicians and nurses applied the intervention in their routine practice in the PHC centre. It consisted of a first screening visit where the PHC professionals assessed the potential risk behaviours. Subsequently, the PHC professionals advised the participant, reached an agreement with them on achievable goals, developed a specific plan taking the three potential risks and stage of change into account, assisted with anticipation of barriers and arranged follow-up support (i.e., 2–3 visits). The duration of the intervention was 12 months and it combined three action levels (i.e., individual, group and community). The individual intervention consisted of a face-to-face intervention to increase awareness of the need for a behaviour change or making a plan for this change. The group intervention consisted of two health education workshops about healthy diet and physical activity while the community intervention consisted of social prescribing of resources available in the catchment area.

Participants in the control PHC centres received usual care, which integrates Spanish preventive protocols involving lifestyle recommendations and preventive activities based on screening and brief advice. These recommendations are focused on lifestyles, cardiovascular and mental diseases, cancer and vaccination [27–31].

### Outcomes and data collection

Information was recorded at baseline and 12-month follow-up.

#### Use of resources, loss of productivity and other costs

Resources included healthcare utilisation in PHC (general practitioner (GP) and nurse home or centre visits, laboratory test, social worker visits, emergency visits) and hospital (admissions and length of admission, emergency visits, diagnostic tests), medication use, sick leave and intervention-related costs such as tobacco use and cost of activities (group activities were managed by a nurse but they varied in attendees and/or duration; cost assumed was one nurse visit at the centre per patient). As described in Supplementary Table, information on healthcare utilisation and sick leave were collected from electronic health records, individual clinical record review and/or a Case Report Form (CRF) depending on the region. Intervention-related cost (tobacco use and cost of activities) were obtained from CRF. The information recorded at baseline referred to the previous year while information recorded at 12-month follow-up referred to the year of the intervention.

#### Unit costs

Healthcare services unit costs were based on public health service tariffs published in Regional Government Official Bulletins, which were updated to 2019 using the specific regional healthcare Consumer Price Index. Subsequently, the mean tariff was calculated for each health service. A social worker visit tariff was not found, and it was assumed to be the same as that of the Nurse centre visit. Medication cost was obtained from administrative databases. The cost of productivity loss was calculated based on the human capital approach using the minimum daily wage in Spain. All costs were expressed in euros 2019. Table 1 shows unit costs used in the study. The time horizon was one year and no discount rate was applied.

**Table 1:** Unit cost (€ in 2019) (mean cost and maximum and minimum regional official tariffs)

	Unit cost		
	Mean	Maximum unit cost	Minimum unit cost
Healthcare perspective			
GP visit in PHCC	55.81	74.80	41.33
GP home visit	77.76	99.47	64.34
Nurse visit in PHCC	25.51	34.51	15.06
Nurse home visit	48.18	80.30	32.06
GP emergency visit	115.42	266.50	62.00
Nurse emergency visit	88.85	278.40	42.92
Social work visit	25.51	34.51	15.06
Other specialist visit	139.15	242.21	54.56
Emergency hospital visit	214.09	375.15	88.77
Hospitalisation (one day of stay)^a^	652.90	925.23	501.62
Laboratory test^b^	49.81	73.66	29.26
Diagnostic test^b^	75.17	180.47	23.38
Medication	Depending on the medication		
Goverment perspective			
Sick leave^c^	34.52	64.78	34.52
Societal perspective			
Tobacco	Detailed by patients		
Community activities			

*GP* General Practitioner; *PHCC* Primary Health Care Centre. ^a^Based on mean tariffs of all hospital complexity types published. ^b^Diagnostic and laboratory test were classified by the research team based on clinical criteria. ^c^Mean and minimum cost in sick leave refer to minimum daily wage while maximum cost refers to mean daily wage

#### Clinical outcomes

The European Quality of Life instrument (EuroQol-5D-3L; EQ5D) was used to measure health-related quality of life [32, 33]. QALYs were calculated by linearly interpolating baseline and one-year follow-up utility score based on EQ5D Spanish tariffs. CVR (expressed in %) was calculated using the REGICOR function chart based on sex, age, total cholesterol, HDL, diagnosis of diabetes, smoker status and blood pressure [34–36]. MHBC, which was the primary outcome of the intervention was defined as a change in at least two unhealthy behaviours. This variable was recorded by the research team in the CRF and, lately, it was dichotomized between change in at least two unhealthy behaviours and no change or change in only one unhealthy behaviour.

Other sociodemographic and clinical variables including participant age, gender, civil status, education level, diagnosis of hypertension, the presence of comorbidities and Body Mass Index (BMI) was also recorded on the CRF.

### Statistical analysis

We assumed that the variables with missing values are Missing At Random (MAR). MAR assumption can be made more plausible by collecting more explanatory variables and including them in the analysis, and we have included almost all the possible explanatory variables (excluding duplicate variables, very similar variables and highly correlated variables to avoid collinearity). Imputation was used to deal with missing data. Missing values varied from 0 to 13% at baseline and from 1 to 44% at follow-up. Multiple imputation by chained equations (MICE) using 50 imputed databases was applied to all variables. This number of the database was based on the fraction of missing information. To perform MICE, we used predictive mean matching in continuous variables, logistic regression in dichotomous variables and polytomous regression in categorical variables (more than two categories). Societal perspective included all costs recorded while healthcare perspective included all costs except productivity losses and tobacco costs. Cost-utility in terms of extra cost per QALY gain and cost-effectiveness in terms of extra cost per a one-point reduction in CVR or per MHBC in one extra participant was estimated by obtaining incremental cost-utility (ICUR) and incremental cost-effectiveness ratios (ICER), respectively. ICUR and ICER were calculated as the difference in the cost between the intervention and the control group, divided by the difference in QALYs, the difference in % CVR points and the probability of modifying behaviours. The base-case analysis was an intention-to-treat analysis. Differences in cost and effects were estimated using adjusted generalised linear models with gamma (for QALYs and cost) or Gaussian (CVR) distributions and estimating marginal means using *margins* STATA command and adjusted logistic regression for behaviour change. These adjustments consisted of baseline cost or effects and those variables that showed statistically significant differences at baseline (i.e., BMI) as was indicated in the study protocol [37]. All analyses clustered participants in PHC centres using the “vce” STATA option. We used bootstrapping with 1000 replications (20 replications in each of the 50 imputed databases) to assess uncertainty and to construct the cost-utility planes and acceptability curves.

We performed six sensitivity analyses: 1) a complete-case analysis (only considering those participants who attended the follow-up visit); 2) an analysis considering mean wage for productivity losses; 3) an analysis considering the maximum regional tariff for each of the healthcare services and 4) the minimum; 5) an analysis adjusted only by baseline costs and effects (not adjusted by BMI) and 6) an analysis using seemingly unrelated regressions. The first four sensitivity analyses were pre-specified [11].

Imputation analyses were performed with R and all other analyses were performed with Stata MP 13.1.

## Results

**Fig. 1:**
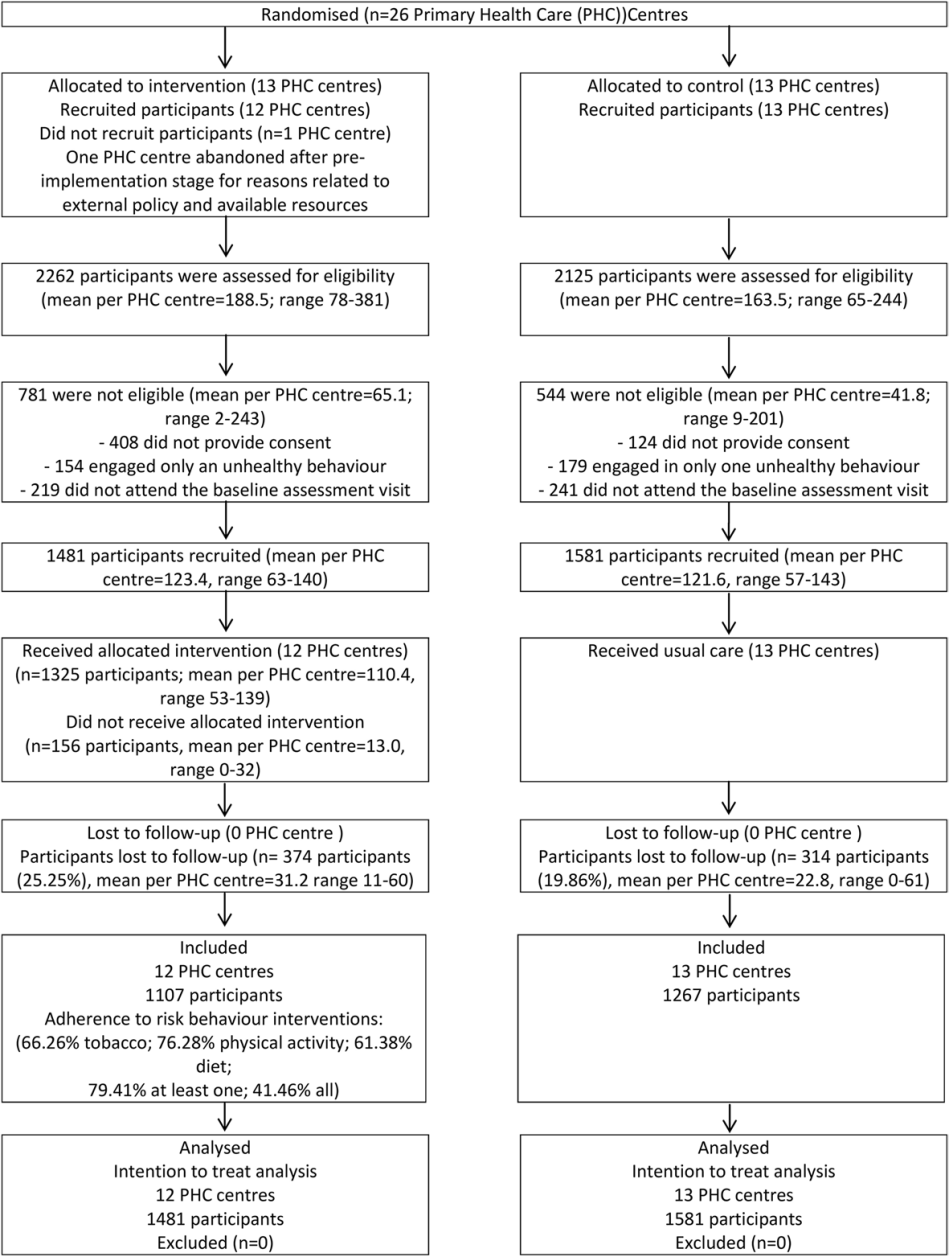
Flow diagram of clusters and participants through study. PHC, Primary Health Care

Overall, 3062 participants (1481 in the intervention group) were included in the study. Figure 1 shows the study flowchart and intervention adherence rates. Lost participants in the follow-up assessment were around 22%. Some 41% of intervention participants were adherent to all MHBC interventions (79% to at least one). Table 2 presents the sample characteristics. There were statistically significant differences between groups at baseline in the BMI, with a higher proportion of the control group in the overweight category and a higher proportion of the intervention group in the obese category.

**Table 2:** Sociodemographic and clinical characteristics of the sample

N (%)	Control Group (*N* = 1581)	Intervention Group (*N* = 1481)	TOTAL (*N* = 3062)	% of missing values
**Women**	872 (55.15)	809 (54.63)	1681 (54.90)	0
**Age, mean; SD**	58.29; 8.31	57.74; 7.88	58.03; 8.11	0
**Birth place**				0.91
Spain	1479 (93.55)	1369 (92.44)	2848 (93.01)	
Other countries	97 (6.14)	89 (6.01)	186 (6.07)	
**Civil Status**				0.85
Married/co-habiting	1055 (66.73)	1024 (69.14)	2079 (67.90)	
Other civil status	520 (32.89)	437 (29.51)	957 (31.25)	
**Education level**				0.95
Secondary or university studies	888 (56.17)	820 (55.37)	1708 (55.78)	
Primary or lower studies	684 (43.26)	641 (43.28)	1325 (43.27)	
**Diabetes**	323 (20.43)	277 (18.70)	600 (19.60)	0.46
**Smoker**	697 (44.09)	641 (43.28)	1338 (43.70)	0
**Hypertension**	646 (40.86)	627 (42.34)	1273 (41.57)	1.27
**Systolic arterial pressure (mmHg), mean; SD**	131.74; 18.30	133.10; 16.33	132.39; 17.40	3.56
**Diastolic arterial pressure (mmHg), mean SD**	80.41; 10.68	82.37; 10.26	81.35; 10.53	3.59
**Total Cholesterol (mg/dL), mean; SD**	205.67; 38.79	205.97; 42.49	205.82; 40.63	8.20
**High-density lipoprotein (mg/dL), mean; SD**	53.48; 14.24	52.79; 14.41	53.15; 14.33	13.29
**Body Mass Index, mean; SD**	**29.27**; **5.64**	**30.62**; **5.85**	29.92; 5.78	1.11
**Adherence to Mediterranean diet (PREDIMED range 0 -- 13), mean; SD**	6.79; 1.99	6.74; 1.97	6.77; 1.98	0.20
**Presence of comorbidities**	935 (59.14)	8760 (58.07)	1795 (58.62)	7.32
**Cardiovascular risk (REGICOR range 0.50 - 30.51), mean; SD**	4.92; 3.63	5.01; 3.41	4.96.; 3.53	15.16
**Health-related quality of life (EQ5D range − 0.07 – 1), mean; SD**	0.82; 0.19	0.81; 0.20	0.82; 0.19	1.5
**Specific risks**				
Tobacco	697 (44.09)	638 (43.08)	1335 (43.60)	0
Diet	1482 (93.74)	1384 (93.45)	2866 (93.60)	0
Physical activity	1448 (91.59)	1345 (90.82)	2793 (91.21)	0

Bold figures indicate statistical differences between intervention and control patients

### Cost-utility and cost-effectiveness of EIRA intervention

The intervention mean cost, including individual visits, workshops and community activities, was €49.33 per person (minimum €0 and maximum €1188). Table 3 shows each mean cost by group at baseline and follow-up and the unadjusted difference between groups at each point. Table 4 shows unadjusted costs for both groups at baseline and follow-up. The intervention effectively promoted MHBC. There were no statistically significant differences between groups in costs, QALYs or cardiovascular risk in the main analyses. Tables 5 and 6 shows the results of the cost-utility and cost-effectiveness analyses. Unadjusted effects for both groups are detailed in Table 4.

**Table 3:** Mean group cost at baseline and follow-up and difference between groups at both points

	Total costs at baseline (95% CI)			Total costs at follow-up (95% CI)		
	**Control**	**Intervention**	**Diff**^**a**^	**Control**	**Intervention**	**Diff** ^**a**^
Healthcare perspective						
GP visit in PHCC	380.44 (361.74; 399.15)	330.20 (313.98; 346.42)	**−50.24 (−74.38; −26.11)**	292.28 (277.21; 307.36)	305.48 (285.75; 325.22)	13.20 (−10.01; 36.41)
GP home visit	37.42 (−32.78; 107.63)	41.48 (−35.06; 118.01)	4.05 (−11.79; 19.90)	31.72 (− 27.61; 91.07)	35.43 (−35.86; 106.71)	3.70 (−11.42; 18.83)
Nurse visit in PHCC	77.12 (69.67; 84.56)	62.48 (56.54; 68.42)	**−14.63 (−24.01; −5.26)**	80.89 (70.28; 91.49)	78.15 (66.95; 89.36)	−2.73 (−16.10; 10.63)
Nurse home visit	14.95 (−16.52; 46.43)	19.70 (−15.98; 55.39)	4.75 (−4.27; 13.78)	17.21 (−16.34; 50.76)	22.94 (−16.09; 61.96)	5.73 (−3.36; 14.81)
GP emergency visit	76.82 (−34.69; 188.34)	87.42 (−37.48; 212.32)	10.60 (−18.58; 39.78)	125.57 (− 43.92; 295.07)	141.38 (−29.57; 312.32)	15.80 (−26.37; 27.97)
Nurse emergency visit	101.29 (− 177.93; 380.51)	102.03 (− 209.28; 413.35)	0.75 (−59.45; 60.94)	155.61 (− 161.78; 473.00)	129.96 (− 159.40; 419.33)	−25.64 (− 105.60; 54.31)
Social work visit	7.32 (−12.08; 26.73)	5.04 (− 5.42; 15.51)	−2.28 (−12.69; 8.12)	7.72 (− 10.12; 25.55)	5.05 (− 5.22; 15.32)	−2.67 (− 11.09; 5.75)
Other specialist visit	277.49 (248.90; 306.08)	286.29 (192.31; 380.26)	8.80 (−81.89; 99.48)	217.55 (187.69; 247.40)	196.26 (56.54; 335.99)	−21.29 (− 149.80; 107.23)
Emergency hospital visit	95.44 (84.17; 106.71)	109.10 (70.49; 147.71)	13.66 (−22.94; 50.26)	66.37 (55.73; 77.01)	89.59 (30.24; 148.93)	23.22 (−31.28; 77.71)
Hospitalisation	181.21 (118.15; 244.27)	280.87 (−86.03; 647.76)	99.66 (− 243.36; 433.68)	368.23 (174.78; 561.69)	516.06 (− 564.99; 1597.11)	147.83 (− 836.51; 1132.16)
Laboratory test	138.58 (125.82; 151.34)	112.16 (103.75; 120.57)	**−26.43 (−41.91; −10.94)**	86.98 (76.20; 97.76)	78.86 (70.84; 86.89)	−8.12 (− 21.68; 5.45)
Diagnostic test	113.02 (101.68; 124.35)	99.84 (89.78; 109.90)	−13.18 (−28.39; 2.03)	73.17 (61.16; 85.19)	63.19 (56.16; 70.22)	−9.98 (−24.12; 4.16)
Medication	592.34 (−51.55; 1236.23)	626.45 (−345.15; 1598.23)	34.20 (− 231.60; 299.99)	682.08 (− 90.79; 1454.95)	768.14 (− 379.34; 1915.62)	86.06 (− 239.10; 411.21)
Goverment perspective						
Sick leave	425.23 (341.00); 509.45)	368.96 (287.50; 450.41)	−56.27 (− 166.91; 54.37)	356.51 (280.57; 432.45)	404.07 (292.65; 515.48)	47.56 (−72.83; 167.94)
Societal perspective						
Tobacco	556.41 (134.76; 978.06)	594.57 (187.79; 1001.34)	38.15 (−62.61; 138.92)	689.04 (−490.48; 1868.57)	698.39 (−598.83; 1995.61)	9.35 (− 122.35; 141.04)
Community activities	251.69 (− 679.02; 1182.40)	255.70 (− 514.40; 1025.81)	4.01 (− 57.54; 65.57)	260.26 (− 595.66; 1116.17)	221.12 (− 304.42; 746.65)	−39.14 (− 129.38; 51.10)

*GP* General Practitioner; *PHCC* Primary Health Care Centre. Bold letters refer to statistically significant differences between groups. ^a^ Differences were obtained based on unadjusted linear regression where the independent variable was the intervention/control group and the dependent variable was the cost of each service/sick leave

**Table 4:** Unadjusted cost at baseline and follow-up and effects for intervention and control patients for the main and sensitivity analysis

	Baseline costs control (95% CI)	Baseline costs intervention (95%CI)	Follow-up costs control (95% CI)	Follow-up costs intervention (95%CI)	QALY control (95% CI)	QALY intervention (95%CI)	% in change in two or three behaviours control (95% CI)	% in change in two or three behaviours intervention (95% CI)	REGICOR control (95% CI)	REGICOR intervention (95%CI)
Main analysis - Societal perspective (ITT and minimum wage)	3317.81 (2472.88; 4162.74)	3371.50 (2184.26; 4558.75)	3509.14 (2097.21; 4921.07)	3719.10 (1701.58 5736.63)	0.82 (0.78; 0.86)	0.80 (0.75; 0.85)	8.95 (5.89; 12.01)	14.50 (10.53; 18.46)	5.32 (3.72; 6.92)	5.28 (3.64; 6.92)
Main analysis – Healthcare system perspective (ITT and minimum wage)	2264.69 (1554.81; 2974.58)	2321.81 (1240.24; 3403.37)	2342.46 (1382.32; 3302.62)	2480.86 (735.35; 4226.37)	0.82 (0.78; 0.86)	0.80 (0.75; 0.85)	8.95 (5.89; 12.01)	14.50 (10.53; 18.46)	5.32 (3.72; 6.92)	5.28 (3.64; 6.92)
Mean wage	3699.53 (2843.41; 4555.64)	3705.86 (4892.79)	3823.72 (2407.10; 5240.34)	4119.24 (2026.52; 6211.97)	0.82 (0.78; 0.86)	0.80 (0.75; 0.85)	8.95 (5.89; 12.01)	14.50 (10.53; 18.46)	5.32 (3.72; 6.92)	5.28 (3.64; 6.92)
Maximum regional tariffs^a^	4215.72 (3169.72; 5261.71)	4267.83 (5814.35; 5721.32)	4382.76 (2717.33; 6048.20)	4543.97 (2099.43; 6988.51)	0.82 (0.78; 0.86)	0.80 (0.75; 0.85)	8.95 (5.89; 12.01)	14.50 (10.53; 18.46)	5.32 (3.72; 6.92)	5.28 (3.64; 6.92)
Minimum regional tariffs	2650.33 (1836.93; 3463.73)	2693.01 (1578,15; 3807.87)	2840.45 (1536.42; 4144.47)	3031.95 (1243.45 4820.46)	0.82 (0.78; 0.86)	0.80 (0.75; 0.85)	8.95 (5.89; 12.01)	14.50 (10.53; 18.46)	5.32 (3.72; 6.92)	5.28 (3.64; 6.92)
Complete-case	3285.49 (2480.49; 4090.49)	3438,17 (2275.87; 4600.47)	3396.87 (2246.36; 4547.365)	3556.06 (1827.95; 5284.16)	0.83 (0.82; 0.84)	0.81 (0.80; 0.82)	5.70 (4.38; 7.02)	11.56 (9.62; 13.51)	4.69 (4.37; 5.00)	4.97 (4.67; 5.27)
SUR	3317.81 (2472.88; 4162.74)	3371.50 (2184.26; 4558.75)	3509.14 (2097.21; 4921.07)	3719.10 (1701.58 5736.63)	0.82 (0.78; 0.86)	0.80 (0.75; 0.85)	8.95 (5.89; 12.01)	14.50 (10.53; 18.46)	5.32 (3.72; 6.92)	5.28 (3.64; 6.92)

^a^Minimum daily wage is maintained as unit cost for sick leave in this sensitivity analysis

**Table 5:** Difference in cost and effects; ICUR and ICER between intervention and control patients for the main and sensitivity analyses based on adjusted models

	Cost difference (95% CI) in €	QALY difference (95% CI)	ICUR (€/QALY)	Difference in change in two or three behaviours in one patient (95% CI)^**d**^	ICER (€/extra change in two or three behaviours in one patient)	REGICOR reduction difference (95% CI)	ICER (€/REGICOR reduction)
Main analysis - Societal perspective (ITT and minimum wage)	295.02 (− 1402.75; 1992.78)	− 0.009 (− 0.03; 0.01)	Dominated	**5.27 (2.59; 7.78)**	5598	0.17 (-0.40; 0.74)	1,727
Main analysis – Healthcare system perspective (ITT and minimum wage)	207.20 (− 1901.24; 2315.63)	− 0.009 (− 0.03; 0.01)	Dominated	**5.27 (2.59; 7.78)**	3932	0.17 (-0.40; 0.74)	1,231
Mean wage (Societal perspective)	433.19 (− 1487.19; 2354.10)	− 0.009 (− 0.03; 0.01)	Dominated	**5.27 (2.59; 7.78)**	8220	0.17 (-0.40; 0.74)	2,536
Maximum regional tariffs^a^	266.19 (− 2405.15 2507.52)	− 0.009 (− 0.03; 0.01)	Dominated	**5.27 (2.59; 7.78)**	5051	0.17 (-0.40; 0.74)	1,559
Minimum regional tariffs	271,64 (− 1137.75; 1681.02)	−0.009 (− 0.03; 0.01)	Dominated	**5.27 (2.59; 7.78)**	6377	0.17 (-0.40; 0.74)	1,590
Complete-case	126.76 (−1.535.45; 1788.98)	−0.009 (− 0.024; 0.006)	Dominated	**5.68 (3.41;7.99)**	2224	0.24 (-0.24; 0.71)	531
Unadjusted analysis^b^	343.60 (− 1406.84; 2094.05)	−0.011 (− 0.032; 0.011)	Dominated	4.47 (2.82; 8.09)	7690	0.15 (-0.41; 0.72)	2,226
SUR	129.82 (− 16.79; 276.43)^c^	−0.008 (− 0.016; − 0.001)	Dominated	NA	NA	0.17 (-0.01; 0.35)	760

All sensitivity analyses considered societal perspective. SUR: Seemingly unrelated regressions. Dominated: Intervention was more costly and less effective. Bold letters refer to statistically significant differences between groups. ^a^ Minimum daily wage is maintained as unit cost for sick leave in this sensitivity analysis. ^b^ Only adjusted by baseline costs or effects. ^c^Confidence interval in cost when CVR is consider as effect is (− 16.21; 275.85). ^d^Confidence interval calculated based on bootstrapping. NA: Not applicable due to the outcome not being a continuous variable

**Table 6:** Difference in cost and effects (change in two or three behaviours and cardiovascular risk); ICER between intervention and control patients and Relative Value Index (RVI) for the main and sensitivity analyses based on adjusted models

	**Usual Care follow-up Cost (95% CI) in €**	**Usual care % Change in two or three in one patient (95% CI)**	**ICER (€/extra change in two or three behaviours in one patient)**	**RVI**	**Usual care REGICOR at follow-up (95% CI)**	**ICER (€/REGICOR reduction)**	**RVI**
Main analysis - Societal perspective (ITT and minimum wage)	3,509.14 (2,097.21; 4,921.07)	8.95 (5.89; 12.01)	5598	0.07	5.32 (3.72; 6.92)	1,727	0.38
Main analysis – Healthcare system perspective (ITT and minimum wage)	2,342.46 (1,382.32; 3,302.62)	8.95 (5.89; 12.01)	3932	0.07	5.32 (3.72; 6.92)	1,231	0.36
Mean wage (Societal perspective)	3,823.72 (2,407.10; 5,240.34)	8.95 (5.89; 12.01)	8220	0.05	5.32 (3.72; 6.92)	2,536	0.28
Maximum regional tariffs^a^	4,382.76(2,717.33; 6,048.20)	8.95 (5.89; 12.01)	5051	0.10	5.32 (3.72; 6.92)	1,559	0.53
Minimum regional tariffs	2,840.45 (1,536.42; 4,144.47)	8.95 (5.89; 12.01)	6377	0.05	5.32 (3.72; 6.92)	1,590	0.34
Complete-case	3,396.87 (2,246.36; 4,547.365)	5.70 (4.38; 7.02)	2224	0.27	4.69 (4.37.; 5.00)	531	1.36
Unadjusted analysis^b^	3,509.14 (2,097.21; 4,921.07)	8.95 (5.89; 12.01)	7690	0.05	5.32 (3.72; 6.92)	2,226	0.30
SUR	3,509.14 (2,097.21; 4,921.07)	NA	NA	NA	5.32 (3.72; 6.92)	760	0.22

All sensitivity analyses considered societal perspective. SUR: Seemingly unrelated regressions. ^a^ Minimum daily wage is maintained as unit cost for sick leave in this sensitivity analysis. ^b^ Only adjusted by baseline costs or effects. ^c^Confidence interval in cost when CVR is consider as effect is (-1.58; 261.22). ^d^Confidence interval calculated based on bootstrapping. NA: Not applicable due to the outcome not being a continuous variable

**Fig. 2:**
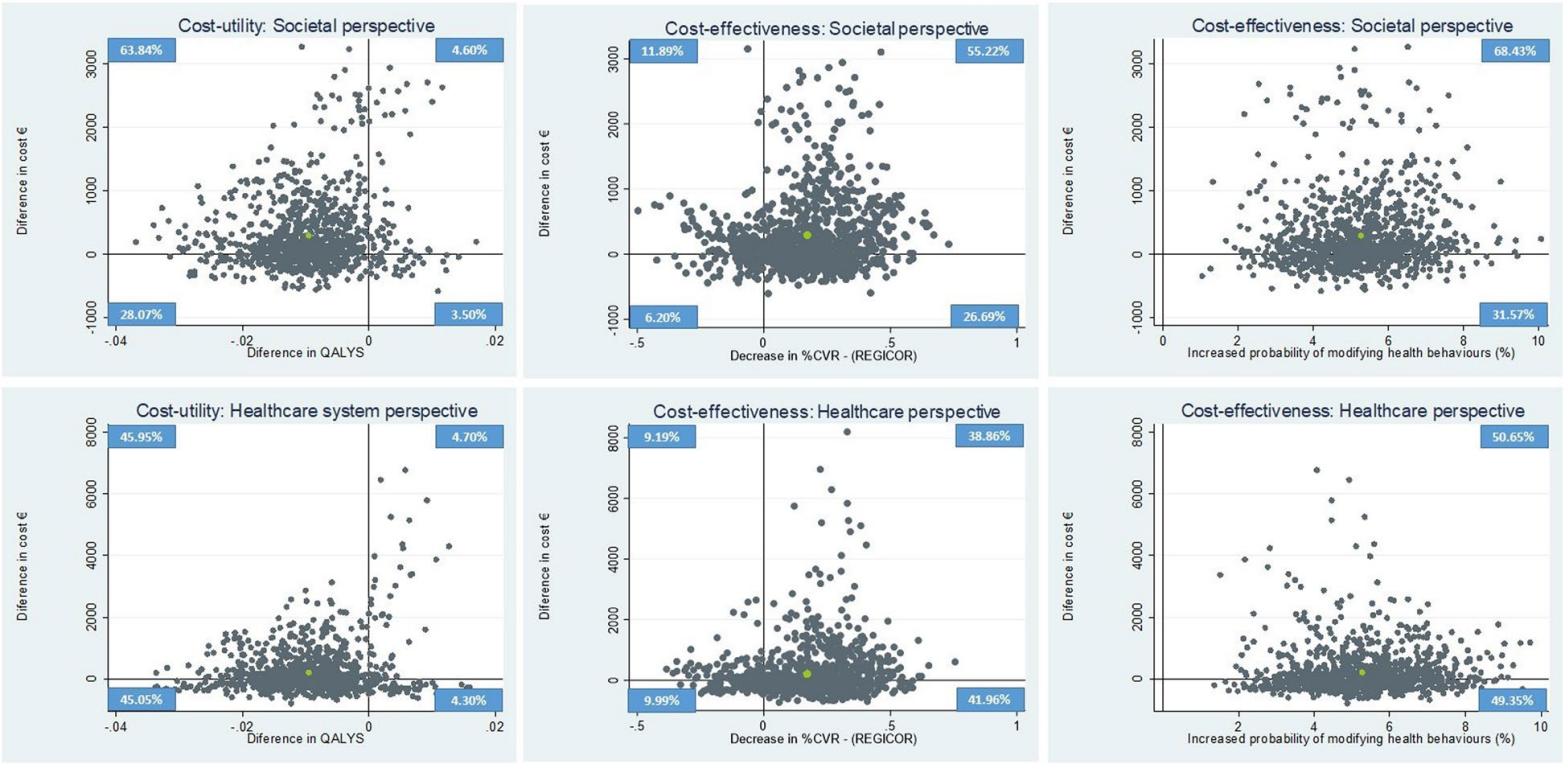
Cost–utility and cost-effectiveness of EIRA intervention vs usual care

Usual care dominated (decreased cost and is more effective) the intervention in the cost-utility analysis. When MHBC is considered in the cost-effectivity analysis, ICERs from the societal and healthcare perspectives were €5,598 and €3,932 per additional change in one patient, respectively. Considering the cardiovascular risk, ICERs from the societal and healthcare perspectives were €1,727 and €1,231 per one-point reduction in cardiovascular risk, respectively. Figure 2 shows cost-utility and cost-effectiveness planes. Cost-utility planes showed high uncertainty surrounding the ICUR. Most bootstrapped incremental cost-utility pairs fell in the north-west (intervention increased costs and is less effective than usual care) or south-west quadrants (intervention decreased costs and is less effective than usual care) of the cost-utility plane. In terms of cardiovascular risk reduction and MHBC, the intervention fell in the north-east (intervention increased costs and is more effective than usual care) and south-east (intervention decreased costs and is more effective than usual care) quadrants of the cost-utility place, although, in the healthcare perspective, most pairs are distributed over the cost axis (€0). The intervention was cost-saving in 32 and 49% of the pairs from the societal and healthcare perspectives while it was more effective in 9% of the pairs in terms of QALYS; 81% of the pairs in terms of CVR and all pairs in terms of MHBC. Acceptability curves are shown in Supplementary Files.

### Sensitivity analysis

Table 5 shows the results of sensitivity analysis. All sensitivity analyses showed similar results to those of the main analyses. The scenarios where the differences in cost were smaller were the complete case and that using seemingly unrelated regressions. The scenario with the largest differences in cost was that considering the mean wage as unit cost for sick leave. In terms of cost-effectiveness, the best scenario for both outcomes was the complete case (ICER per extra MHBC in one participant was €2,224 while ICER per REGICOR reduction was €531), while the worse scenario was that considering the mean wage as unit cost for sick leave.

Table 4 also shows the unadjusted mean cost and effects of the sensitivity analysis.

## Discussion

### Summary

The cost-effectiveness of the EIRA intervention measured in terms of MHBC remains unclear. However, although the intervention was shown to be no more costly than usual care and it promoted MHBC, the probabilistic analysis showed high uncertainty surrounding cost differences and intervention did not affect quality of life while cardiovascular risk reduction was limited. Based on the results of this economic evaluation, the EIRA intervention should be reformulated before being translated to clinical practice or it should be assessed with a longer follow-up period.

### Strengths and limitations

The large, representative sample is the main strength of this study. This was a multisite study evaluating an MHBC intervention implemented in a complex context such as PHC. Participants from 25 PHC centres in 7 Spanish regions were included and this represents one of the first economic evaluations that has been developed under these circumstances. The pragmatism of this trial is one of the key points of its design as it was developed with the aim of an immediate implementation in the PHC system throughout the modification and elimination of potential barriers. We intended to develop a flexible intervention that could be adapted to different PHC settings, and the intervention’s design was based on the results of previous phases of the study. These features led to adherence being measured in a permissive way and the content of the intervention itself in each of the centres was not fully documented. This fact together with a low fidelity of the intervention could have limited our capacity to detect differences between groups [38]. Similarly, not all cost information was gathered using the same protocols (each region has combined electronic health record download, individual clinical record review and/or CRF based on availability) and this may introduce some bias. However, it increased the external validity of the results of the study. The follow-up period may have limited the impact of the intervention in outcomes such as QALYs and CVR reduction considering that intervention is focused on health promotion.

### Comparison with existing literature

There were no differences in costs between groups. As suggested previously, health promotion interventions have a very low cost or, at least, a similar cost compared to usual care [7, 8, 39–41]. Similarly, the intervention had no effect on QALYs at one-year follow-up. Although QALYs are the standard effect measure in cost-utility analysis, health promotion interventions are not expected to produce improvements in QALYs in the short term. Similar results have been observed in economic evaluations of health promotion interventions in PHC [7, 8, 39–42]. However, some of these studies showed positive ICURs with a clear dominance of the intervention [7, 40]. Although the effect of these interventions was small (0.01 for QALYS and 80£ for costs [7] or 0.02 for QALYS and €-16 for cost [40]), the uncertainty regarding the cost-effectiveness of the intervention was also small. The ICUR dominance in some of these preventive interventions could be related to the targeted population (obese) [7] or the risk approached (major depression) [40] while the EIRA intervention is focused on health behaviours in a general population sample where differences in QALYs after 1 year is hardly seen.

MHBC was more sensitive to the intervention in the short term. The EIRA intervention presented statistical differences in this outcome and showed a societal ICER of €5600 per one extra patient changing two or three unhealthy behaviours. This ICER was reduced to €3900 when the healthcare perspective was considered. Previous studies showed lower ICERs, up to £986 (€1098 at November 2020), per one extra patient behaviour change [8, 10]. However, most of these interventions only focus on one behaviour. Although previous authors have suggested proposals of transforming unit changes of different outcomes into comparable metrics, currently, there is no national or international threshold in terms of behaviour change to consider an intervention efficient. In any case, the EIRA intervention would seem to exceed this virtual threshold; especially when the national threshold to consider an intervention efficient in terms of QALYs is €20,000 per QALYs [43] and given the difficulty in translating healthy behaviours into gains in QALYs [44]. A suggested alternative to thresholds is the Relative Value Index, being the formula for this index: RVI = (usual care mean costs/usual care mean effects)/ICER)) [45]. A Relative Value Index greater than 1 means that a new intervention would provide better outcomes at a lower incremental cost per outcome than the comparator. However, values below 1 and close to 0, as EIRA intervention shows (taking into account mean cost and effects, in terms of MHBC, in the usual care group) would mean *that the additional outcome associated with the new intervention would cost more than the previously “accepted value” of a cost per outcome*.

Partially although similar results were in the same line observed when CVR was considered as the outcome, ICER was more affordable. The EIRA intervention showed a societal ICER of €61,727900 per one-point reduction in CVR and a healthcare ICER of 41,231900. In this situation, although this extra cost could seem reasonable, the lack of evidence and recommendations about willingness to pay for reduction in CVR hinders the interpretation and subsequent recommendations. and considering that usual care already involves preventive protocols, it is very difficult to observe substantial changes in these outcomes, and consequently, CVR in the short-medium term. Furthermore, the real impact of the intervention could be larger because changes in the medium-long term can be preceded by promotion interventions on healthy lifestyles which have an impact on CVR [46].

The largest effect in CVR and MHBC was observed in the complete-case analysis; this might suggest that higher levels of intervention fidelity would have produced better results. However, this effect is still clearly lower than previous preventive PHC interventions aiming to reduce CVR [47], other related outcomes such as weight [7], or behaviour change [8, 10].

A technical but crucial issue is the model and assumption choice. In this paper, we considered generalized linear models as the main choice due to previous recommendations related to checking non-normality distribution in cost and effects after a visual inspection [37]. However, it is important to emphasise that seemingly unrelated regression, a choice that led to correlate the error terms across regression models in cost and effects but assuming normality, was the second most favourable scenario to the intervention in terms of cost-utility.

### Implication for research and practice

In a globalized world, it is increasingly important to develop complex healthcare actions that can be adapted to each patient and implemented in a large number of settings. At this point, developing hybrid trials is a very good option. This is particularly important in the context of PHC where systemic interventions predominate and professionals approach more than one health or risk behaviour. However, researchers should not overlook the importance of monitoring the specific actions in each setting. This would ensure that clear information is available to determine whether these actions are genuinely (cost)effective or not.

Developing cost-effective interventions that promote healthy lifestyles are crucial to support the healthcare system. A healthy population should be an optimal target for these interventions although, due to patient health status, intervention assessment needs to be extended into the medium-long term. Conducting economic models which assess the long-term cost-effectiveness using mortality and QALYS as outcomes would give relevant information for health decision-makers. Similarly, standardizing the use of other generalizable outcomes that could be sensitive to health promotion interventions would help decision making in this field. A healthy population is usually located in very heterogeneous and complex contexts, a fact that complicated the registry and cost definition, key factors for economic evaluations. In this line, efforts should be taken in standardization without leaving the complexity of the context out of consideration [48].

## Conclusions

A health promotion PHC intervention such as EIRA effectively impact MHBC in the Spanish context but its efficiency cannot be fully established under current circumstances. The recommendation of this intervention should be conditioned by positive results in the medium-long term clinical assessment or by modification of the intervention.

## Supplementary Information

**Additional file 1: Supplementary Figure.** Acceptability curves for cost-utility analysis.

**Additional file 2: Supplementary Table.** Source of information of cost and effects.

## Data Availability

The data that support the findings of this study are available from IDIAP Jordi Gol but restrictions apply to the availability of these data, which were used under license for the current study, and so are not publicly available. Data are however available from the authors upon reasonable request and with permission of IDIAP Jordi Gol.

## Abbreviations

MHBC: Multiple health behaviour changes
QALY: Quality-adjusted life years
ICUR: Cost-utility ratio
ICER: Incremental cost-effectiveness ratio
PHC: Primary health care
CVR: Cardiovascular risk
GP: General practitioner
CRF: Case report form
EQ5D: EuroQol-5D-3L
BMI: Body mass index
