# Correction: Cost-effectiveness analysis of a multiple health behaviour change intervention in people aged between 45 and 75 years: a cluster randomized controlled trial in primary care (EIRA study)

**DOI:** 10.1186/s12966-024-01674-8

**Published:** 2024-12-09

**Authors:** Ignacio Aznar-Lou, Edurne Zabaleta-Del-Olmo, Marc Casajuana-Closas, Alba Sánchez-Viñas, Elizabeth Parody-Rúa, Bonaventura Bolíbar, Montserrat Iracheta-Todó, Oana Bulilete, Tomàs López-Jiménez, Haizea Pombo-Ramos, María Victoria Martín Miguel, Rosa Magallón-Botaya, Jose Ángel Maderuelo-Fernández, Emma Motrico, Juan Bellón, Ruth Martí-Lluch, Maria Rubio-Valera, Antoni Serrano-Blanco

**Affiliations:** 1 Research and development Unit, Parc Sanitari Sant Joan de Déu, Institut de Recerca Sant Joan de Déu, Dr. Antoni Pujades 42, 08830 Sant Boi de Llobregat, Barcelona, Catalonia Spain; 2 Consortium for Biomedical Research in Epidemiology & Public Health (CIBER en Epidemiología y Salud Pública - CIBERESP), Madrid, Spain; 3 Fundació Institut Universitari per a la recerca a l’Atenció Primària de Salut Jordi Gol i Gurina (IDIAPJGol), Barcelona, Spain; 4 Gerència Territorial de Barcelona, Institut Català de la Salut, Barcelona, Spain; 5 Departament d’InfermeriaFacultat d’Infermeria, Universitat de Girona, Girona, Spain; 6 Universitat Autònoma de Barcelona, Bellaterra, Cerdanyola del Vallès, Spain; 7 Primary Care Prevention and Health Promotion Network (redIAPP), Palma de Mallorca, Spain; 8 Primary Care Research Unit, Mallorca, Balearic Public Health Service, Palma de Mallorca, Spain; 9 Health Research Institute of the Balearic Islands (IdISBa), Palma de Mallorca, Spain; 10 Primary Care Research Unit of Bizkaia, Basque Health Service-Osakidetza, Bilbao, Spain; 11 Biocruces Bizkaia Health Research Institute, Barakaldo, Bizkaia Spain; 12 Vigo Primary Health Care, Vigo, Spain; 13 I-Saúde Research Group (IISGS), Vigo, Spain; 14 IIS-Aragón Grupo b21-17R, Universidad de Zaragoza, Zaragoza, Spain; 15 CS Arrabal.Servicio Aragonés de Salud, Zaragoza, Spain; 16 Primary Health Care Research Unit of Salamanca (APISAL), Health Service of Castilla y León (SACyL), Institute of Biomedical Research of Salamanca (IBSAL), Salamanca, Spain; 17 Universidad Loyola Andalucía, Sevilla, Spain; 18 Centro de Salud El Palo, Málaga, Spain; 19 Department of Preventive Medicine, University of Málaga, Málaga, Spain; 20 Biomedical Research Institute of Malaga (IBIMA), Málaga, Spain; 21 ISV Research Group, Research Unit in Primary Care, Primary Care Services, Girona, Catalan Institute of Health (ICS), Girona, Catalonia Spain; 22 Biomedical Research Institute, Girona (IdIBGi), ICS, Girona, Catalonia Spain

**Correction** **:** **Int J Behav Nutr Phys Act 18, 88 (2021)**

**https://doi.org/10.1186/s12966-021-01144-5**

After publication of this article, the authors identified a miscalculation in the cardiovascular risk data. As a result, certain sections of the article, including text, Tables 2, 4, 5, 6, and Fig. 2 need to be updated.

The corrected text, along with the affected rows and columns of the tables and the revised Fig. 2 are given below.

The original article has been corrected.

**Table:** 

**Section**	**Page**	**Incorrect**	**Correct**
Abstract (Results)	2	Differences in QALYS or cardiovascular risk between-group were close to 0 (-0.01 and 0.04 respectively). The ICER was €5,598 per extra health behaviour change in one patient and €6,926 per one-point reduction in cardiovascular risk from a societal perspective.	Differences in QALYS or cardiovascular risk between-group were close to 0 (-0.01 and 0.17 respectively). The ICER was €5,598 per extra health behaviour change in one patient and €1,727 per one-point reduction in cardiovascular risk from a societal perspective.
Results (Cost-utility and cost-effectiveness of EIRA intervention)	6	When MHBC is considered in the cost-effectivity analysis, ICERs from the societal and healthcare perspectives were €5598 and €3932 per additional change in one patient, respectively. Considering the cardiovascular risk, ICERs from the societal and healthcare perspectives were €6926 and €4864 per one-point reduction in cardiovascular risk, respectively.	When MHBC is considered in the cost-effectivity analysis, ICERs from the societal and healthcare perspectives were €5,598 and €3,932 per additional change in one patient, respectively. Considering the cardiovascular risk, ICERs from the societal and healthcare perspectives were €1,727 and €1,231 per one-point reduction in cardiovascular risk, respectively.
Results (Sensitivity analysis)	11	The scenario with the largest differences in cost was that considering the mean wage as unit cost for sick leave. In terms of cost effectiveness, the best scenario for both outcomes was the complete case (both ICER were around €2200 per extra MHBC in one participant or REGICOR reduction), while the worse scenario was that considering the mean wage as unit cost for sick leave.	The scenario with the largest differences in cost was that considering the mean wage as unit cost for sick leave. In terms of cost-effectiveness, the best scenario for both outcomes was the complete case ( ICER per extra MHBC in one participant was €2,224 while ICER per REGICOR reduction was €531), while the worse scenario was that considering the mean wage as unit cost for sick leave
Discussion (Summary)	11	The cost-effectiveness of the EIRA intervention measured in terms of MHBC remains unclear. However, although the intervention was shown to be no more costly than usual care and it promoted MHBC, the probabilistic analysis showed high uncertainty surrounding cost differences and intervention did not affect quality of life or cardiovascular risk reduction.	The cost-effectiveness of the EIRA intervention measured in terms of MHBC remains unclear. However, although the intervention was shown to be no more costly than usual care and it promoted MHBC, the probabilistic analysis showed high uncertainty surrounding cost differences and intervention did not affect quality of life while cardiovascular risk reduction was limited
Discussion (Comparison with existing literature)	12	Partially similar results were observed when CVR was considered as the outcome. The EIRA intervention showed a societal ICER of €6900 per one-point reduction in CVR and a healthcare ICER of 4900. In this situation, and considering that usual care already involves preventive protocols, it is very difficult to observe substantial changes in these outcomes, and consequently, CVR in the short-medium term. Furthermore, changes in the medium-long term can be preceded by promotion interventions on healthy lifestyles which have an impact on CVR [46].	Partially although similar results were in the same line observed when CVR was considered as the outcome, ICER was more affordable. The EIRA intervention showed a societal ICER of €61,727900 per one-point reduction in CVR and a healthcare ICER of 41,231900. In this situation, although this extra cost could seem reasonable, the lack of evidence and recommendations about willingness to pay for reduction in CVR hinders the interpretation and subsequent recommendations. and considering that usual care already involves preventive protocols, it is very difficult to observe substantial changes in these outcomes, and consequently, CVR in the short-medium term. Furthermore, the real impact of the intervention could be larger because changes in the medium-long term can be preceded by promotion interventions on healthy lifestyles which have an impact on CVR [46].

**Table 2:** Sociodemographic and clinical characteristics of the sample

**N (%)**	**Control Group (N=1,581)**	**Intervention Group (N=1,481)**	**TOTAL (N=3,062)**	**% of missing values**
**Cardiovascular risk (REGICOR range 0.50 – 30.51), mean; SD**	4.92; 3.63	5.01; 3.41	4.96.; 3.53	15.16

**Table 4:** Unadjusted cost at baseline and follow-up and effects for intervention and control patients for the main and sensitivity analysis.

	**REGICOR control (95% CI)**	**REGICOR intervention (95%CI)**
Main analysis - Societal perspective (ITT and minimum wage)	5.32 (3.72; 6.92)	5.28 (3.64; 6.92)
Main analysis – Healthcare system perspective (ITT and minimum wage)	5.32 (3.72; 6.92)	5.28 (3.64; 6.92)
Mean wage	5.32 (3.72; 6.92)	5.28 (3.64; 6.92)
Maximum regional tariffs^a^	5.32 (3.72; 6.92)	5.28 (3.64; 6.92)
Minimum regional tariffs	5.32 (3.72; 6.92)	5.28 (3.64; 6.92)
Complete-case	4.69 (4.37.; 5.00)	4.97 (4.67; 5.27)
SUR	5.32 (3.72; 6.92)	5.28 (3.64; 6.92)

**Table 5:** Difference in cost and effects; ICUR and ICER between intervention and control patients for the main and sensitivity analyses based on adjusted models.

	**REGICOR reduction difference (95% CI)**	**ICER (€/REGICOR reduction)**
Main analysis - Societal perspective (ITT and minimum wage)	0.17 (-0.40; 0.74)	1,727
Main analysis – Healthcare system perspective (ITT and minimum wage)	0.17 (-0.40; 0.74)	1,231
Mean wage (Societal perspective)	0.17 (-0.40; 0.74)	2,536
Maximum regional tariffs^a^	0.17 (-0.40; 0.74)	1,559
Minimum regional tariffs	0.17 (-0.40; 0.74)	1,590
Complete-case	0.24 (-0.24; 0.71)	531
Unadjusted analysis^b^	0.15 (-0.41; 0.72)	2,226
SUR	0.17 (-0.01; 0.35)	760

All sensitivity analyses considered societal perspective. SUR: Seemingly unrelated regressions. Dominated: Intervention was more costly and less effective. ^a^Minimum daily wage is maintained as unit cost for sick leave in this sensitivity analysis. ^b^Only adjusted by baseline costs or effects. ^c^Confidence interval in cost when CVR is consider as effect is (-16.21; 275.85). ^d^Confidence interval calculated based on bootstrapping. NA: Not applicable due to the outcome not being a continuous variable

**Table 6:** Difference in cost and effects (change in two or three behaviours and cardiovascular risk); ICER between intervention and control patients and Relative Value Index (RVI) for the main and sensitivity analyses based on adjusted models

	**Usual Care follow-up Cost (95% CI) in €**	**Usual care % Change in two or three in one patient (95% CI)**	**ICER (€/extra change in two or three behaviours in one patient)**	**RVI**	**Usual care REGICOR at follow-up (95% CI)**	**ICER (€/REGICOR reduction)**	**RVI**
Main analysis - Societal perspective (ITT and minimum wage)	3,509.14 (2,097.21; 4,921.07)	8.95 (5.89; 12.01)	5598	0.07	5.32 (3.72; 6.92)	1,727	0.38
Main analysis – Healthcare system perspective (ITT and minimum wage)	2,342.46 (1,382.32; 3,302.62)	8.95 (5.89; 12.01)	3932	0.07	5.32 (3.72; 6.92)	1,231	0.36
Mean wage (Societal perspective)	3,823.72 (2,407.10; 5,240.34)	8.95 (5.89; 12.01)	8220	0.05	5.32 (3.72; 6.92)	2,536	0.28
Maximum regional tariffs^a^	4,382.76 (2,717.33; 6,048.20)	8.95 (5.89; 12.01)	5051	0.10	5.32 (3.72; 6.92)	1,559	0.53
Minimum regional tariffs	2,840.45 (1,536.42; 4,144.47)	8.95 (5.89; 12.01)	6377	0.05	5.32 (3.72; 6.92)	1,590	0.34
Complete-case	3,396.87 (2,246.36; 4,547.365)	5.70 (4.38; 7.02)	2224	0.27	4.69 (4.37.; 5.00)	531	1.36
Unadjusted analysis^b^	3,509.14 (2,097.21; 4,921.07)	8.95 (5.89; 12.01)	7690	0.05	5.32 (3.72; 6.92)	2,226	0.30
SUR	3,509.14 (2,097.21; 4,921.07)	NA	NA	NA	5.32 (3.72; 6.92)	760	0.22

All sensitivity analyses considered societal perspective. SUR: Seemingly unrelated regressions. ^a^Minimum daily wage is maintained as unit cost for sick leave in this sensitivity analysis. ^b^Only adjusted by baseline costs or effects. ^c^Confidence interval in cost when CVR is consider as effect is (-1.58; 261.22). ^d^Confidence interval calculated based on bootstrapping. NA: Not applicable due to the outcome not being a continuous variable

**Fig. 2:**
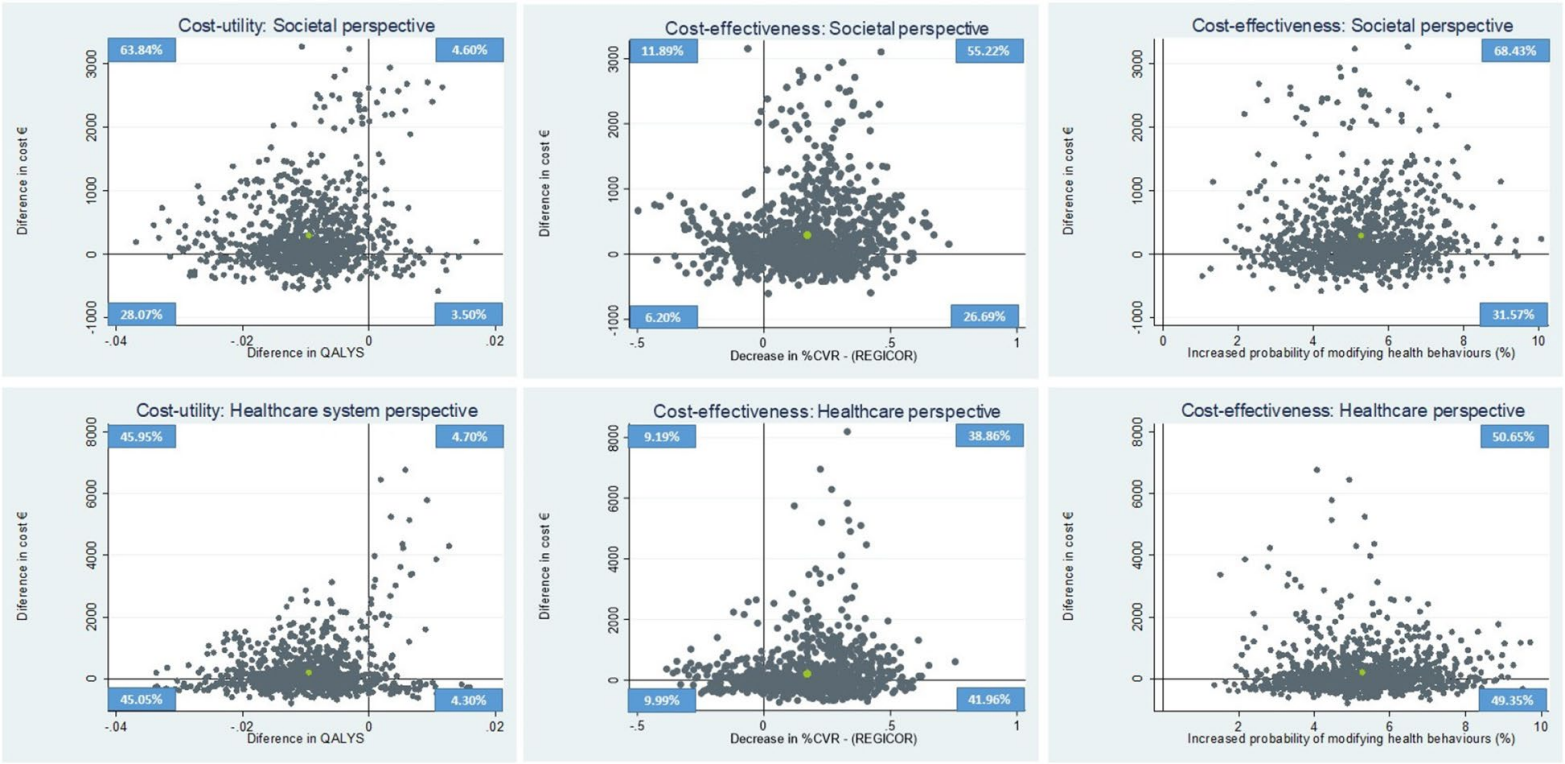
Cost–utility and cost-effectiveness of EIRA intervention vs usual care

