# Supplementary material for: Cost-effectiveness analysis of a multiple health behaviour change intervention in people aged between 45 and 75 years: a cluster randomized controlled trial in primary care (EIRA study)
Source: Int J Behav Nutr Phys Act. 2021 Jul 2;18:88. doi: 10.1186/s12966-021-01144-5 (PMC8254273; doi:10.1186/s12966-021-01144-5)
Supplement: Supplementary file 1 — Additional file 1: Supplementary Figure. Acceptability curves for cost-utility analysis. [file 12966_2021_1144_MOESM1_ESM.docx]

Supplementary Figure. Acceptability curves for cost-utility analysis.

Acceptability curves for cost-effectiveness analysis in terms of extra cost per change in two or more unhealthy behaviours.

Acceptability curves for cost-effectiveness analysis in terms of extra cost per reduction in one cardiovascular risk percentage point.
