# Supplementary material for: Cost-effectiveness analysis of a multiple health behaviour change intervention in people aged between 45 and 75 years: a cluster randomized controlled trial in primary care (EIRA study)
Source: Int J Behav Nutr Phys Act. 2021 Jul 2;18:88. doi: 10.1186/s12966-021-01144-5 (PMC8254273; doi:10.1186/s12966-021-01144-5)
Supplement: Supplementary file 2 — Additional file 2: Supplementary Table. Source of information of cost and effects. [file 12966_2021_1144_MOESM2_ESM.docx]

**Supplementary Table**. Source of information of cost and effects

|  | **AND** | **ARG** | | **CyL** | **CAT** | **BI** | | **GAL** | **BC** |
| --- | --- | --- | --- | --- | --- | --- | --- | --- | --- |
| Primary Care |  |  | |  |  |  | |  |  |
| Secondary Care |  |  | |  |  |  | |  |  |
| Medication |  |  | |  |  |  | |  |  |
| Sick leave |  |  | |  |  |  | |  |  |
| Intervention |  |  | |  |  |  | |  |  |
| Health related quality of Life (EQ5D) |  |  | |  |  |  | |  |  |
| **Electronic health records** | | | **Individual clinical record review** | | | | **Case Report form** | | |

AND: Andalucia; ARG: Aragon; CyL: Castile and Leon; CAT: Catalonia; BI: the Balearic Islands; GAL: Galicia; BC: Basque Country; EQ5D: EuroQol-5D-3L
